# Brain Invasion in Meningioma—A Prognostic Potential Worth Exploring

**DOI:** 10.3390/cancers13133259

**Published:** 2021-06-29

**Authors:** Felix Behling, Johann-Martin Hempel, Jens Schittenhelm

**Affiliations:** 1 Department of Neurosurgery, University Hospital Tübingen, Eberhard-Karls-University Tübingen, 72076 Tübingen, Germany; 2 Center for CNS Tumors, Comprehensive Cancer Center Tübingen-Stuttgart, University Hospital Tübingen, Eberhard-Karls-University Tübingen, 72076 Tübingen, Germany; johann-martin.hempel@uni-tuebingen.de (J.-M.H.); jens.schittenhelm@med.uni-tuebingen.de (J.S.); 3 Department of Diagnostic and Interventional Neuroradiology, University Hospital Tübingen, Eberhard-Karls-University Tübingen, 72076 Tübingen, Germany; 4 Department of Neuropathology, University Hospital Tübingen, Eberhard-Karls-University Tübingen, 72076 Tübingen, Germany

**Keywords:** meningioma, invasive growth, brain invasion, prognosis, recurrence, WHO classification for CNS tumors

## Abstract

**Simple Summary:**

Meningiomas are benign tumors of the meninges and represent the most common primary brain tumor. Most tumors can be cured by surgical excision or stabilized by radiation therapy. However, recurrent cases are difficult to treat and alternatives to surgery and radiation are lacking. Therefore, a reliable prognostic marker is important for early identification of patients at risk. The presence of infiltrative growth of meningioma cells into central nervous system tissue has been identified as a negative prognostic factor and was therefore included in the latest WHO classification for CNS tumors. Since then, the clinical impact of CNS invasion has been questioned by different retrospective studies and its removal from the WHO classification has been suggested. There may be several reasons for the emergence of conflicting results on this matter, which are discussed in this review together with the potential and future perspectives of the role of CNS invasion in meningiomas.

**Abstract:**

Most meningiomas are slow growing tumors arising from the arachnoid cap cells and can be cured by surgical resection or radiation therapy in selected cases. However, recurrent and aggressive cases are also quite common and challenging to treat due to no established treatment alternatives. Assessment of the risk of recurrence is therefore of utmost importance and several prognostic clinical and molecular markers have been established. Additionally, the identification of invasive growth of meningioma cells into CNS tissue was demonstrated to lead to a higher risk of recurrence and was therefore integrated into the WHO classification of CNS tumors. However, the evidence for its prognostic impact has been questioned in subsequent studies and its exclusion from the next WHO classification proposed. We were recently able to show the prognostic impact of CNS invasion in a large comprehensive retrospective meningioma cohort including other established prognostic factors. In this review we discuss the growing experiences that have been gained on this matter, with a focus on the currently nonuniform histopathological assessment, imaging characteristics and intraoperative sampling as well as the overall outlook on the future role of this potential prognostic factor.

## 1. Introduction

Meningiomas account for approximately one third of primary intracranial tumors [1]. Based on the increasing availability of cranial imaging the incidence seems to rise constantly [2]. They arise from arachnoid cap cells and usually show slow circumscribed growth and are therefore considered benign neoplasms [3]. However, about 20% show a more aggressive behavior with an increased proliferation rate and a higher tendency to recur [3]. Most meningiomas can be treated effectively by surgical excision, while radiation therapy is reserved for selected cases, recurrent tumors and primarily fast-growing subtypes [4,5,6,7]. There are no other established therapy options and especially, more aggressive meningiomas are challenging to treat. After multiple rounds of resection and radiation there is no established treatment alternative to oppose further disease progression, while individualized treatment approaches are frequently offered [8].

During the last decade, a central part of meningioma research was the identification and description of potential markers to improve the prognostic assessment of patients. On a molecular level, tumors can be roughly dichotomized as *NF2* (neurofibromatosis type 2) and non-*NF2*-mutated meningiomas, and *NF2* is the main identified driver mutation in atypical meningiomas [9,10]. Mutations of the promotor of the telomere reverse transcriptase (TERT), loss of the cyclin-dependent kinase inhibitor 2A/B (CDKN2A/B) and loss of the trimethylation of lysin 27 of histone 3 (H3K27me3), were identified as negative prognostic markers independent of tumor histology [11,12,13,14].

Brain invasion in meningiomas involves a complex interplay between cell adhesion molecules, extracellular matrix, the basement membrane at the brain tumor interface and the stimulation of growth factors. For further details of a three-step process that involves degradation, migration and differentiation the reader is referred to the review by Qin et al. [15]. In proteomic analysis the Wnt signaling cascade was identified as one of the significantly modulated pathways in meningioma and is involved in epithelial-to-mesenchymal transition [16]. It is likely that brain invasion through epithelial-to-mesenchymal transition may be modulated by altered expression of beta-catenin and E-cadherin in meningiomas as members of the Wnt pathway [17,18].

With the introduction of the updated WHO classification for central nervous system tumors in 2016, the detection of CNS invasion was integrated as a stand-alone criterion for atypia, leading to a WHO grade II assignment in tumors without other signs of atypia [3]. Since then, the prognostic role of brain invasion in meningioma has been questioned because of the contradictory results of different retrospective analyses [19,20,21]. It is currently under discussion, whether it should be removed from the upcoming WHO classification or whether the diagnosis should be supplemented with an appropriate reference to the contradictory data situation. However, there is an obvious problem regarding tumor sampling, which is usually done in a non-standardized way, potentially minimizing the chance for neuropathologists to detect invasive growth into CNS tissue. Furthermore, no clear-cut criteria exist for the histopathological detection of invasive growth into brain parenchyma and the supporting staining. Another important aspect, which has attracted little attention so far, is the potential of preoperative imaging regarding the illustration of invasive features, which would be of great help for preoperative planning.

Overall, the evidence on the role of CNS invasion is scarce but promising. In this review, the role and potential of CNS invasion in meningioma is illustrated from three different points of view: histopathological, imaging and intraoperative assessment.

## 2. Histopathological Assessment of Invasive Growth

In 2016, brain invasion in meningioma was clearly defined as an additional criterion for atypia in the revised fourth edition of the WHO classification of central nervous system tumors. Such tumors, even in the absence of mitotic activity—so called otherwise histologically benign meningiomas—are now classified as WHO grade II tumors [3]. The supporting evidence is based on a single comprehensive study of 89 cases, wherein CNS parenchyma was present within the tumor specimen. For recurrence-free analysis 22 cases were classified as brain invasive and compared to 54 cases with the absence of brain invasion [22]. Brain invasion was defined by Perry et al. as fingerlike or knobby protrusions into underlying cortex and/or absence of a leptomeningeal layer at the tumor-CNS interface. The previous classification in 2007 used the same study as the main evidence and suggested that such tumors “should thus prognostically be considered as WHO grade II” [23], resulting in divergent tumor grading in various neuropathological institutions between 2007 and 2016. The same study was also referenced in the third edition of the WHO classification of 2000 to indicate a negative prognostic significance for brain invasion. However, the classification of 2000 did not recommend this as a criterion for atypia [24]. Therefore, brain invasive meningiomas were usually assigned WHO grade I between 2000 and 2007 [23,24]. Previously, the second edition of WHO classification in 1993 mentioned brain invasion as a malignancy criterion only among anaplastic (grade III) meningiomas [25]. In the same year, McLean and colleagues questioned the role of brain invasion for tumor grading because they found no prognostic role in 28 meningiomas [26]. Their analysis was restricted to grade II and III meningiomas and did not include otherwise histologically benign meningiomas. A follow-up series in 1999 by Perry et al. addressed this question directly and examined 20 meningiomas (only 23% of all the brain-invasive meningiomas were otherwise histologically benign after exclusion of subtotally resected tumors due to their higher recurrence risk) showing a recurrence-free survival similar to atypical meningioma [27]. Because Perry et al. observed only 4% brain invasive tumors, he expected that the proposed changes to the WHO classification in 2016 would affect the incidence of WHO grade II tumors by 1%, but was confronted with a “mini-epidemic in brain invasive otherwise benign meningiomas” and suggested that a loosened approach of pathologic criteria to identify brain invasion was the underlying cause [28]. As the concordance level between observers of detecting brain invasion in meningiomas is generally very high (kappa 0.76; highest kappa of all tested features in NRG Oncology RTOG Trial 0539) interobserver histological interpretation differences alone may not explain these discrepancies [29]. The revised 2016 WHO grading resulted in further studies questioning the role of brain invasion in meningiomas. Vranic et al., Streckert et al. and Champeaux et al. found a clear association with histologic brain invasion and recurrence-free survival, but their cases were restricted to grade II/III tumors [30,31,32]. In their cohort of 19 patients with brain invasive otherwise benign meningiomas, Pizem et al. did not observe an increased incidence of recurrence [33]. Spille et al. identified 20 patients with brain invasive otherwise benign meningiomas that showed recurrence rates similar to WHO grade I tumors [34]. Another retrospective analysis of 49 grade II meningiomas reviewed the prognostic impact of different histopathological criteria. In this series brain invasion showed no difference in the recurrence rate [35]. A larger analysis by Baumgarten et al. compared 149 atypical meningiomas with 61 patients with brain invasive otherwise benign meningiomas and found only four relapses in the latter cohort [19]. A large bi-institutional study including 170 cases with available CNS tissue found no association with progression-free survival and brain invasion [36]. In a retrospective multicenter analysis of 200 atypical meningiomas that were treated with gross total resection, brain invasion was not associated with an increased risk of recurrence [37]. In contrast, a recent bi-institutional study compared 25 otherwise benign meningiomas and 40 atypical brain invasive meningiomas and identified brain invasion as an independent prognostic factor for the progression-free interval [38].

Because of the highly controversial data, the role of brain invasion remains unsolved, and the upcoming fifth edition of the WHO CNS tumor classification attempts to refine the histological criteria for a more stringent classification. While tongue-like protrusions in the absence of intervening leptomeninges remain essential, meningoangiomatosis-like tumor extension along Virchow-Robin spaces do not constitute brain invasion due to an intact pia mater. The diverging results of studies on otherwise histologically benign meningiomas are now noted in the WHO classification and the need for further studies to resolve this issue is mentioned [28]. Because the majority of brain invasive meningiomas also show other atypical features (such as elevated mitotic activity) and brain invasion in grade II tumors is no longer disputed, only a subset of otherwise benign meningiomas is affected by the ongoing controversy. Previous studies have proposed an increased expression of SPARC (secreted protein, acidic and rich in cysteine) and matrix metalloproteinase-9 (MMP-9) as a sign of brain-invasive meningioma in the absence of an infiltrative interface with the brain [39]. In addition, increased expression of CD44 and GFAP in astrocytic processes of CNS tissue adjacent to tumor tissue suggests the absence of brain infiltration due their adhesion to intact basement membrane [40]. GFAP staining is now highly recommended in otherwise histologically benign meningiomas to address brain invasion in borderline cases, but grading of such cases may be superseded in the future by established prognostic molecular markers such as the TERT promotor mutation and/or CDKN2A/B deletion. Standardized tumor sampling in future prospective studies is a prerequisite for obtaining reproducible data regarding brain invasion.

## 3. Imaging Aspects of Invasive Growth

There is no established imaging characteristic that clearly depicts infiltrative growth of meningiomas into the adjacent CNS parenchyma. In order to establish such radiographic criteria, it is necessary to have definite criteria for the histopathological detection of invasive growth as a reference. Furthermore, it would be necessary to have detailed sampling that can be allocated to the preoperative imaging. Since there is no consensus either on the histopathological definition of parenchymal invasion or a standardized sampling algorithm, it is difficult to establish imaging characteristics of invasive growth.

**Figure 1:**
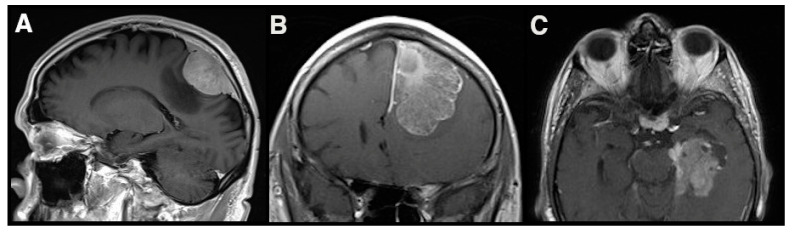
Three meningiomas of the Tübingen Meningioma Cohort are shown as representative examples of MR-Imaging characteristics that have been associated with invasive growth (peritumoral edema (**A**); irregular contrast enhancement (**B**); irregular tumor shape (**C**)). In all three cases histopathological evidence of invasive growth into CNS parenchyma was detected.

However, there are a few studies that have addressed this radiographic aspect. Adeli et al. analyzed imaging characteristics of 617 meningiomas and found an irregular tumor shape, heterogenous contrast enhancement and peritumoral edema to be associated with the detection of brain invasion (examples presented in Figure 1). However, only peritumoral edema remained as an independent predictor of brain invasion in the multivariate analysis [41]. The predictive effect of peritumoral edema was later confirmed in other studies [42,43]. Parenchymal edema adjacent to the meningioma interface seems to be an expression of close interaction of the tumor with bordering structures, such as damage to the cortex by an infiltrating tumor. However, it has to be kept in mind that edema is also associated with meningioma size [44] and can also form, based on the pressure-dependent compromise of parenchymal venous drainage.

The utilization of radiomics has also been studied in meningiomas. A recent retrospective analysis has shown the prognostic potential of preoperative MRI radiomic features in 128 primary grade I meningiomas [45]. Regarding infiltrative growth, Kandemirli et al. compared radiomics-based postcontrast MRI features of 56 meningiomas with ascertained brain invasion and 52 without brain invasion, including grade I and II tumors. The method showed good potential in predicting brain invasion, especially when compared to grade I meningiomas [46]. The most recent study on this topic by Joo et al. analyzed radiomic features in 454 meningiomas, including 88 with histopathologically proven brain invasion. As already mentioned, volume of peritumoral edema was again identified as an independent predictive factor of brain invasion, but furthermore, brain-meningioma interface features were also of significance [42]. Of note, radiomics has also shown potential in predicting bone invasion of meningiomas in preoperative imaging in a series of 490 tumors [47].

Although, imaging characteristics and especially radiomic features show great promise in predicting invasive growth of meningiomas, further studies need to confirm its feasibility. Furthermore, it is of great interest to assess the prognostic impact of radiographic detection of CNS invasion, due to its independence from non-standardized intraoperative tissue sampling compared to histopathological assessment.

## 4. Intraoperative Assessment and the Sampling Dilemma

**Figure 2:**
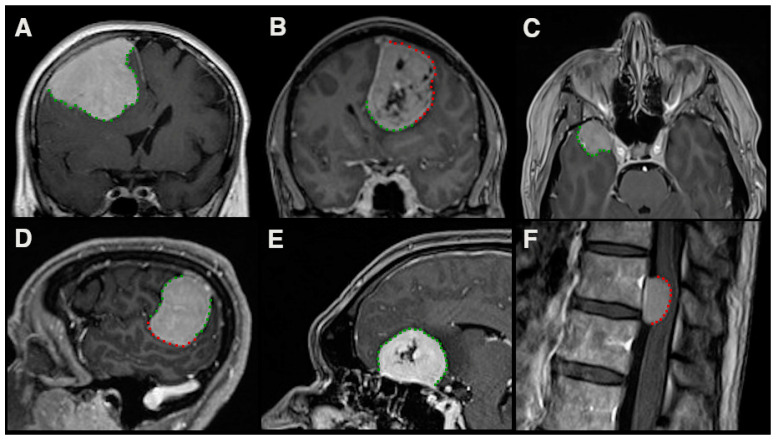
Illustration of the area of interest for meningioma sampling regarding the detection of CNS invasion. Dotted lines represent contact of the meningioma surface with non-eloquent (green) and eloquent (red) parenchyma ((**A**): right frontal convexity meningioma; (**B**): left frontoparietal falcine meningioma; (**C**): right-sided sphenoid wing meningioma; (**D**): left temporo-parietal convexity meningioma; (**E**): olfactory groove meningioma; (**F**): ventral thoracic meningioma).

We recently demonstrated the prognostic potential of the detection of CNS invasion in a retrospective analysis where we compared the prognostic impact of intraoperative and histopathological detection of invasive growth. The combination of both assessment types showed a clear prognostic impact in a comprehensive multivariate analysis. Interestingly, based on the intraoperative surgical evaluation, features of infiltrative growth were observed in 23.7%, and only in 4.8% during histopathological assessment [48]. This reflects the probable inconsistent sampling of areas of interest for histopathological assessment, thus possibly leading to undersampling of CNS invasion. Unless the complete margin between the meningioma and cortical structures is provided for histopathological analysis (see Figure 2), CNS invasion can never be completely ruled out. This may very well have been a decisive factor in other retrospective studies, that were unable to show a prognostic impact of CNS invasion [19,20].

There are several factors than can potentially influence the sampling. One is the tumor location. In cases of skull base meningiomas the resection can be technically demanding, especially when done through key-hole approaches. Such resection techniques are the result of constant refinement of neurosurgical techniques and improvement of visualization via the operative microscope and endoscope over several decades, which has led to less morbidity and improved cosmetic results [49,50,51]. This development has evolved without consideration of possible sampling problems, which have become more evident in light of the possible prognostic role of CNS invasion, only in the last few years. However, performing a larger surgical exposure during meningioma resection in order to possibly improve tumor sampling and taking into account a higher risk of morbidity, is unacceptable in light of the currently uncertain prognostic evidence of CNS invasion.

Furthermore, meningiomas adjacent to highly eloquent areas may be difficult to resect, especially if adhesions or possibly infiltrative growth is present [52]. In such cases an expanded sampling to include bordering cortical tissue would increase the risk of postoperative morbidity. Especially in such areas, it is of utmost importance to respect the cortex even if adhesions or infiltrations are present. In non-eloquent areas it may be possible to include adjacent cortical tissue with the sample in cases of clear intraoperative infiltrative growth. This way meningioma protrusions into parenchyma may be histopathologically detectable. However, it is ethically questionable to expand the sampling to neighboring cortical structures, especially in light of the uncertain prognostic impact of CNS invasion.

Intraoperative labeling of suspicious areas of the tumor sample by the surgeon could be a first step towards improving the assessment of invasive growth [53].

## 5. Outlook

The clinical and prognostic role of invasive growth of meningiomas into central nervous system tissue has the potential to improve prognostication. First of all, it is important to raise awareness in the neurosurgical society of the sampling problem. We recently launched an online survey among members of the German Neurosurgical Society to assess the perception of this issue and if specific measures have been undertaken to improve or standardize meningioma sampling. Thus far, no national/international standards or expert recommendations exist.

Furthermore, factors that hamper a complete tumor sampling need to be identified and may then be further addressed. It is suspected that optimal intraoperative conditions for meningioma sampling will very likely contradict the trend of performing smaller and more focused surgical approaches, especially for skull base meningiomas. It will be challenging to balance and satisfy both extremes, maximum chance for neuropathological detection of CNS invasion as well as minimal invasive neurosurgical approaches for optimal clinical and cosmetic outcomes.

However, most importantly, it is necessary to provide evidence of high quality for the prognostic role of CNS invasion. Without a prospective study, including a clear documentation of the intraoperative evaluation and sampling as well as the histopathological analysis, the evidence for or against the role of CNS invasion will remain questionable. In order to tackle this problem, we are planning a prospective multicenter study.

In case the prognostic impact of CNS invasion becomes more established, the future may bring uniform standards for the intraoperative sampling and histopathological assessment, together with a clearer insight into the incidence and distribution among different meningioma locations. This would be an important basis for the assessment of possible tissue markers that may be associated with invasive growth and could simplify diagnostic efforts and help us provide better care for meningioma patients. There are older reports of potential markers such as SPARC that need to be reassessed in light of the growing potential of CNS invasion and novel molecular markers emerging [54]. A deeper insight into the mechanisms of infiltrative growth can furthermore spark the development of innovative therapeutic approaches. In that respect, extracellular matrix degradation, cell adhesion and growth factors have been formulated as potential therapy targets [15].

Overall, CNS invasion in meningiomas has a high potential to improve the risk stratification for our patients. We highly encourage other researchers to provide their data and experiences in order to advance this highly interesting research area.

## 6. Conclusions

The prognostic significance of CNS invasion in meningioma is currently under discussion. Due to non-uniform histopathological criteria and non-standardized intraoperative sampling, the potential of CNS invasion has not yet been utilized to its full extent.

## References

[1] Ostrom Q.T., Patil N., Cioffi G., Waite K., Kruchko C., Barnholtz-Sloan J.S. (2020). CBTRUS Statistical Report: Primary Brain and Other Central Nervous System Tumors Diagnosed in the United States in 2013–2017. Neuro Oncol..

[2] Spasic M., Pelargos P.E., Barnette N., Bhatt N.S., Lee S.J., Ung N., Gopen Q., Yang I. (2016). Incidental Meningiomas: Management in the Neuroimaging Era. Neurosurg. Clin. N. Am..

[3] Louis D.N., Perry A., Reifenberger G., von Deimling A., Figarella-Branger D., Cavenee W.K., Ohgaki H., Wiestler O.D., Kleihues P., Ellison D.W. (2016). The 2016 World Health Organization Classification of Tumors of the Central Nervous System: A summary. Acta Neuropathol..

[4] Goldbrunner R., Minniti G., Preusser M., Jenkinson M.D., Sallabanda K., Houdart E., von Deimling A., Stavrinou P., Lefranc F., Lund-Johansen M. (2016). EANO guidelines for the diagnosis and treatment of meningiomas. Lancet. Oncol..

[5] Li D., Jiang P., Xu S., Li C., Xi S., Zhang J., Chen Y., Jiang X., Zhang X., Sai K. (2019). Survival impacts of extent of resection and adjuvant radiotherapy for the modern management of high-grade meningiomas. J. Neurooncol..

[6] Nguyen E.K., Nguyen T.K., Boldt G., Louie A.V., Bauman G.S. (2019). Hypofractionated stereotactic radiotherapy for intracranial meningioma: A systematic review. Neurooncol. Pract..

[7] Weber D.C., Ares C., Villa S., Peerdeman S.M., Renard L., Baumert B.G., Lucas A., Veninga T., Pica A., Jefferies S. (2018). Adjuvant postoperative high-dose radiotherapy for atypical and malignant meningioma: A phase-II parallel non-randomized and observation study (EORTC 22042-26042). Radiother. Oncol..

[8] Wilson T.A., Huang L., Ramanathan D., Lopez-Gonzalez M., Pillai P., De Los Reyes K., Kumal M., Boling W. (2020). Review of Atypical and Anaplastic Meningiomas: Classification, Molecular Biology, and Management. Front. Oncol..

[9] Harmanci A.S., Youngblood M.W., Clark V.E., Coskun S., Henegariu O., Duran D., Erson-Omay E.Z., Kaulen L.D., Lee T.I., Abraham B.J. (2017). Integrated genomic analyses of de novo pathways underlying atypical meningiomas. Nat. Commun..

[10] Clark V.E., Erson-Omay E.Z., Serin A., Yin J., Cotney J., Ozduman K., Avsar T., Li J., Murray P.B., Henegariu O. (2013). Genomic analysis of non-NF2 meningiomas reveals mutations in TRAF7, KLF4, AKT1, and SMO. Science.

[11] Behling F., Fodi C., Gepfner-Tuma I., Kaltenbach K., Renovanz M., Paulsen F., Skardelly M., Honegger J., Tatagiba M., International Consortium on Meningiomas (2020). H3K27me3 loss indicates an increased risk of recurrence in the Tubingen meningioma cohort. Neuro Oncol..

[12] Katz L.M., Hielscher T., Liechty B., Silverman J., Zagzag D., Sen R., Wu P., Golfinos J.G., Reuss D., Neidert M.C. (2018). Loss of histone H3K27me3 identifies a subset of meningiomas with increased risk of recurrence. Acta Neuropathol..

[13] Sahm F., Schrimpf D., Olar A., Koelsche C., Reuss D., Bissel J., Kratz A., Capper D., Schefzyk S., Hielscher T. (2016). TERT Promoter Mutations and Risk of Recurrence in Meningioma. J. Natl. Cancer Inst..

[14] Sievers P., Hielscher T., Schrimpf D., Stichel D., Reuss D.E., Berghoff A.S., Neidert M.C., Wirsching H.G., Mawrin C., Ketter R. (2020). CDKN2A/B homozygous deletion is associated with early recurrence in meningiomas. Acta Neuropathol..

[15] Qin C., Huang M., Pan Y., Li Y., Long W., Liu Q. (2021). Brain-invasive meningiomas: Molecular mechanisms and potential therapeutic options. Brain Tumor. Pathol..

[16] Pecina-Slaus N., Kafka A., Lechpammer M. (2016). Molecular Genetics of Intracranial Meningiomas with Emphasis on Canonical Wnt Signalling. Cancers.

[17] Brunner E.C., Romeike B.F., Jung M., Comtesse N., Meese E. (2006). Altered expression of beta-catenin/E-cadherin in meningiomas. Histopathology.

[18] Bukovac A., Kafka A., Raguz M., Brlek P., Dragicevic K., Muller D., Pecina-Slaus N. (2021). Are We Benign? What Can Wnt Signaling Pathway and Epithelial to Mesenchymal Transition Tell Us about Intracranial Meningioma Progression. Cancers.

[19] Baumgarten P., Gessler F., Schittenhelm J., Skardelly M., Tews D.S., Senft C., Dunst M., Imoehl L., Plate K.H., Wagner M. (2016). Brain invasion in otherwise benign meningiomas does not predict tumor recurrence. Acta Neuropathol..

[20] Biczok A., Jungk C., Egensperger R., von Deimling A., Suchorska B., Tonn J.C., Herold-Mende C., Schichor C. (2019). Microscopic brain invasion in meningiomas previously classified as WHO grade I is not associated with patient outcome. J. Neurooncol..

[21] Nakasu S., Nakasu Y. (2021). Prognostic significance of brain invasion in meningiomas: Systematic review and meta-analysis. Brain Tumor. Pathol..

[22] Perry A., Stafford S.L., Scheithauer B.W., Suman V.J., Lohse C.M. (1997). Meningioma grading: An analysis of histologic parameters. Am. J. Surg. Pathol..

[23] Louis D.N., Ohgaki H., Wiestler O.D., Cavenee W.K., Burger P.C., Jouvet A., Scheithauer B.W., Kleihues P. (2007). The 2007 WHO classification of tumours of the central nervous system. Acta Neuropathol..

[24] Kleihues P., Louis D.N., Scheithauer B.W., Rorke L.B., Reifenberger G., Burger P.C., Cavenee W.K. (2002). The WHO classification of tumors of the nervous system. J. Neuropathol. Exp. Neurol..

[25] Kleihues P., Burger P.C., Scheithauer B.W. (1993). The new WHO classification of brain tumours. Brain Pathol..

[26] McLean C.A., Jolley D., Cukier E., Giles G., Gonzales M.F. (1993). Atypical and malignant meningiomas: Importance of micronecrosis as a prognostic indicator. Histopathology.

[27] Perry A., Scheithauer B.W., Stafford S.L., Lohse C.M., Wollan P.C. (1999). “Malignancy” in meningiomas: A clinicopathologic study of 116 patients, with grading implications. Cancer.

[28] Perry A. (2021). The definition and role of brain invasion in meningioma grading: Still controversial after all these years. Free Neuropathol..

[29] Rogers C.L., Perry A., Pugh S., Vogelbaum M.A., Brachman D., McMillan W., Jenrette J., Barani I., Shrieve D., Sloan A. (2016). Pathology concordance levels for meningioma classification and grading in NRG Oncology RTOG Trial 0539. Neuro Oncol..

[30] Vranic A., Popovic M., Cör A., Prestor B., Pizem J. (2010). Mitotic count, brain invasion, and location are independent predictors of recurrence-free survival in primary atypical and malignant meningiomas: A study of 86 patients. Neurosurgery.

[31] Champeaux C., Wilson E., Shieff C., Khan A.A., Thorne L. (2016). WHO grade II meningioma: A retrospective study for outcome and prognostic factor assessment. J. Neurooncol..

[32] Streckert E.M.S., Hess K., Sporns P.B., Adeli A., Brokinkel C., Kriz J., Holling M., Eich H.T., Paulus W., Spille D.C. (2019). Clinical, radiological, and histopathological predictors for long-term prognosis after surgery for atypical meningiomas. Acta Neurochir..

[33] Pizem J., Velnar T., Prestor B., Mlakar J., Popovic M. (2014). Brain invasion assessability in meningiomas is related to meningioma size and grade, and can be improved by extensive sampling of the surgically removed meningioma specimen. Clin. Neuropathol..

[34] Spille D.C., Heß K., Sauerland C., Sanai N., Stummer W., Paulus W., Brokinkel B. (2016). Brain Invasion in Meningiomas: Incidence and Correlations with Clinical Variables and Prognosis. World Neurosurg..

[35] Zima L., Baine M.J., Sleightholm R., Wang B., Punsoni M., Aizenberg M., Zhang C. (2021). Pathologic Characteristics Associated with Local Recurrence of Atypical Meningiomas Following Surgical Resection. J. Clin. Med. Res..

[36] Biczok A., Karschnia P., Vitalini R., Lenski M., Greve T., Thorsteinsdottir J., Egensperger R., Dorn F., Tonn J.C., Schichor C. (2021). Past medical history of tumors other than meningioma is a negative prognostic factor for tumor recurrence in meningiomas WHO grade I. Acta Neurochir..

[37] Fioravanzo A., Caffo M., Di Bonaventura R., Gardiman M.P., Ghimenton C., Ius T., Maffeis V., Martini M., Nicolato A., Pallini R. (2020). A Risk Score Based on 5 Clinico-Pathological Variables Predicts Recurrence of Atypical Meningiomas. J. Neuropathol. Exp. Neurol..

[38] Banan R., Abbetmeier-Basse M., Hong B., Dumitru C.A., Sahm F., Nakamura M., Krauss J.K., Hartmann C. (2021). The prognostic significance of clinicopathological features in meningiomas: Microscopic brain invasion can predict patient outcome in otherwise benign meningiomas. Neuropathol. Appl. Neurobiol..

[39] Backer-Grondahl T., Moen B.H., Arnli M.B., Torseth K., Torp S.H. (2014). Immunohistochemical characterization of brain-invasive meningiomas. Int. J. Clin. Exp. Pathol..

[40] Zeltner L., Schittenhelm J., Mittelbronn M., Roser F., Tatagiba M., Mawrin C., Kim Y.J., Bornemann A. (2007). The astrocytic response towards invasive meningiomas. Neuropathol. Appl. Neurobiol..

[41] Adeli A., Hess K., Mawrin C., Streckert E.M.S., Stummer W., Paulus W., Kemmling A., Holling M., Heindel W., Schmidt R. (2018). Prediction of brain invasion in patients with meningiomas using preoperative magnetic resonance imaging. Oncotarget.

[42] Joo L., Park J.E., Park S.Y., Nam S.J., Kim Y.H., Kim J.H., Kim H.S. (2021). Extensive peritumoral edema and brain-to-tumor interface MRI features enable prediction of brain invasion in meningioma: Development and validation. Neuro Oncol..

[43] Ong T., Bharatha A., Alsufayan R., Das S., Lin A.W. (2021). MRI predictors for brain invasion in meningiomas. Neuroradiol. J..

[44] Simis A., de Aguiar P.H.P., Leite C.C., Santana P.A., Rosemberg S., Teixeira M.J. (2008). Peritumoral brain edema in benign meningiomas: Correlation with clinical, radiologic, and surgical factors and possible role on recurrence. Surg. Neurol..

[45] Ko C.C., Zhang Y., Chen J.H., Chang K.T., Chen T.Y., Lim S.W., Wu T.C., Su M.Y. (2021). Pre-operative MRI Radiomics for the Prediction of Progression and Recurrence in Meningiomas. Front. Neurol..

[46] Kandemirli S.G., Chopra S., Priya S., Ward C., Locke T., Soni N., Srivastava S., Jones K., Bathla G. (2020). Presurgical detection of brain invasion status in meningiomas based on first-order histogram based texture analysis of contrast enhanced imaging. Clin. Neurol. Neurosurg..

[47] Zhang J., Sun J., Han T., Zhao Z., Cao Y., Zhang G., Zhou J. (2020). Radiomic features of magnetic resonance images as novel preoperative predictive factors of bone invasion in meningiomas. Eur. J. Radiol..

[48] Behling F., Fodi C., Gepfner-Tuma I., Machetanz K., Renovanz M., Skardelly M., Bornemann A., Honegger J., Tabatabai G., Tatagiba M. (2020). CNS Invasion in Meningioma-How the Intraoperative Assessment Can Improve the Prognostic Evaluation of Tumor Recurrence. Cancers.

[49] Andrews R.J., Bringas J.R. (1993). A review of brain retraction and recommendations for minimizing intraoperative brain injury. Neurosurgery.

[50] Banu M.A., Mehta A., Ottenhausen M., Fraser J.F., Patel K.S., Szentirmai O., Anand V.K., Tsiouris A.J., Schwartz T.H. (2016). Endoscope-assisted endonasal versus supraorbital keyhole resection of olfactory groove meningiomas: Comparison and combination of 2 minimally invasive approaches. J. Neurosurg..

[51] Borghei-Razavi H., Truong H.Q., Fernandes-Cabral D.T., Celtikci E., Chabot J.D., Stefko S.T., Wang E.W., Snyderman C.H., Cohen-Gadol A., Gardner P.A. (2018). Minimally Invasive Approaches for Anterior Skull Base Meningiomas: Supraorbital Eyebrow, Endoscopic Endonasal, or a Combination of Both? Anatomic Study, Limitations, and Surgical Application. World Neurosurg..

[52] Raffa G., Picht T., Scibilia A., Rosler J., Rein J., Conti A., Ricciardo G., Cardali S.M., Vajkoczy P., Germano A. (2019). Surgical treatment of meningiomas located in the rolandic area: The role of navigated transcranial magnetic stimulation for preoperative planning, surgical strategy, and prediction of arachnoidal cleavage and motor outcome. J. Neurosurg..

[53] Jenkinson M.D., Santarius T., Zadeh G., Aldape K.D. (2017). Atypical meningioma-is it time to standardize surgical sampling techniques?. Neuro Oncol..

[54] Rempel S.A., Ge S., Gutierrez J.A. (1999). SPARC: A potential diagnostic marker of invasive meningiomas. Clin. Cancer Res..

